# Intra-renal pressure and temperature during ureteroscopy: Does it matter?

**DOI:** 10.1590/S1677-5538.IBJU.2020.0428

**Published:** 2021-02-03

**Authors:** Antonio Corrêa Lopes, Vinícius Dall’Aqua, Raphael V. Carrera, Wilson R. Molina, Sidney Glina

**Affiliations:** Faculdade de Medicina do ABC Divisão de Endourologia Departamento de Urologia Santo Andre SP Brasil Departamento de Urologia, Divisão de Endourologia, Faculdade de Medicina do ABC, Santo Andre, SP, Brasil; Endourology Group Kansas University Medical Center Department of Urology Kansas US Department of Urology, Endourology Group Kansas University Medical Center, Kansas, US

## INTRODUCTION

Kidney stone prevalence has raised up in past few years (1-3). Several factors contributed to this growth rate including professional journey, dietetics habits and metabolic syndrome including obesity and Diabetes Mellitus (4, 5). Moreover, urolithiasis is a chronic disease with a recurrence rate close to 50% in 5 years (6, 7).

Frequently, urolithiasis treatment requires surgical intervention. Endoscopic approach is the gold standard and retrograde intra-renal surgery (RIRS) and semi-rigid ureteroscopy are the most common procedures performed for kidney and ureteral stones removal (8, 9). New technology including digital and disposable flexible ureteroscopes, well designed guidewires, baskets, access sheaths and high-power laser systems with small fibers are particularly interesting devices to guarantee safety and attractivity for retrograde approach. Ureteroscopy’s complications are rare (10), but urothelial lesions, peri-renal collections, infection with or without sepsis can be related to surgical technique.

Ureteroscopy is normally performed with fluid irrigation and intra-renal temperature and pressure must be taken in consideration due the fact it may correlate to complications, especially sepsis risk.

Our paper aims to review ureteroscopy surgical aspects regarding intra-renal pressure/temperature and the relationship between this parameters and potential complication involved.

### Intra-renal pressure

Physiological intra-renal pressure ranges from 5 to 10cm H2O and it is increased during endoscopic surgery. Some studies reported 40cm H2O as the safe upper cut off for intra-operative pressure (11). Complication rate increases in higher intra-renal pressure scenarios. Saline irrigation influx control and ureteral access sheath use are the main determinants to avoid the increasing in intra-renal pressure during ureteroscopy.

Saline irrigation plays a major role during ureteroscopy, leading to ureteral lumen expansion and permitting renal pelvis and calices visualization and ureteroscope free navigation through the entire collecting system. Historically, ureteroscopes first generations were not equipped with a robust working channel and no irrigation was possible. Intra-operative diuretics were commonly used to minimize the effects of irrigation deficit (12). Upgrade in ureteroscopes technology added irrigation devices for working channels and improved surgeon visualization. However, saline flow can potentially increase intra-renal pressure (11, 13). As previously mentioned, pressure of 40cm of H2O or higher can increase post-operative complication risks, including bleeding, perirenal collection, and sepsis (14). Sepsis is probably explained by pyelo-venous reflux mechanism, translocating bacteria from urinary colleting system into the blood stream.

There are several ways to use saline to irrigate during ureteroscopy and gravity irrigation is the simplest one. As another way is to use manual pumps; recently, modern advanced irrigation system (Thermedx^®^ FluidSmartTM) has been produced and utilized to better control flow and intraluminal pressure controls during endoscopic procedures, especially percutaneous nephrolithotomy. Noureldin et al. showed that when gravity flow irrigation was used, ureteral and intra-pelvic pressures were low and within a safe range. When manual pump was utilized, intra-pelvic pressures reached peaks of 84 and 105mmH2O, increasing complication risks during the procedure (15).

Flexible ureteroscopes typically have only one working channel, used simultaneously for irrigation and endoscopic tools use, such as baskets, guidewires, and laser fibers. It is well-known that when the working channel has an endoscopic tool inside, saline flow decreases considerably, and stone visualization is compromised (16). Williams et al. also studied flexible ureteroscope geometrical aspects and its influence in irrigation flow rates. They found that scope deflection does not reduce irrigation flow significantly: totally deflected ureteroscope showed only 5% of flow decrease. Moreover, oval shaped working channel seems to delivery better irrigation flow compared to circular ones. These findings might be helpful for future ureteroscopes generation to improve irrigation performance (17). As irrigation is essential for a clean surgical field view during ureteroscopy, temporary intra-operative visualization impairment due to bleeding, stone fragments and even poor scopes quality can lead surgeons to increase irrigation flow rates to enhance visualization, increasing intra-renal pressure and rising potential complications events. Auge et al. showed that intra-renal pressure was increased when ureteroscope is inside the renal pelvis (18).

Schawalb et al., in a porcine model, evaluated intra-enal pressure dynamics and kidney morphological changes during ureteroscopy. Significant saline backflow through the renal parenchyma was observed in intra-renal pressures higher than 30cmH2O. It also caused caliceal urothelium flattening and erosion associated with vacuolization, degeneration, and glomerular hemorrhage. Renal cortex histological analysis after 4-6 weeks demonstrated focal scarring in tested kidneys, whereas no scarring was encountered in low pressures operated kidneys. Tubular histology effects were significantly more traumatic in the group submitted to higher intra-renal pressures (combined to an increased incidence of tubular and collecting ducts dilation, as well as interstitial inflammation). In the same study, authors showed that intra-renal pressure was reduced by keeping bladder empty. Renal pressure increases by 20 to 25mmHg when bladder is fulfilled, supporting the use of a urethral catheter during ureteroscopy (14).

There are other ways to decrease intra-renal pressure during ureteroscopy. Jung et al. studied the effects of intraluminal β-agonist to reduce intra-renal pressure (19). In this trial, use of saline irrigation containing isoproterenol, a non-selective β-agonist, decreased endoluminal pressure (20). Despite these results, use of intra-operative β-agonist has never been incorporated in the best clinical practice for endoscopic urological procedure. On the other hand, use of ureteral access sheaths became popular. Several studies showed that the use of ureteral access sheath dramatically reduces intra-renal pressure during retrograde intra-renal surgery. Access sheath use associated to smaller ureteroscope diameters are paramount to keep intra-renal pressure below the 40cmH2O safety threshold (11). During ureteroscopy, intra-renal pressure is lower closer to proximal ureteral access sheath than in ureteroscope distal extremity (21).

Ureteral access sheaths (UAS) were developed by Takayasu et al. to made easier both ureteral and renal access, serving as a guide tube (22). Recent improvements in UAS were incorporated as different diameters, lengths, and materials. Use of access sheath is not mandatory; however, many studies have demonstrated its benefits such as easier navigation and decreased intra-renal pressure during surgery, explained by better saline outflow (23, 24). Kourambas et al. proposed that operative time and costs could be reduced using UAS (25). On the other hand, Traxer et al. demonstrated no benefits in operative time when ureteral access sheath was employed. However, this study lacked randomization and the UAS group had a larger stone burden (26). UAS use is a good alternative to optimize fluid irrigation and promotes reduced intra-renal pressures. Irrigation levels can be increased by 35 to 80% when a ureteral access sheath is utilized, keeping the same intra-renal pressure (21). Auge et al. showed that regardless ureteroscope style or ureteral access sheath position (located in the ureter or renal pelvis), intra-renal pressure reduction can reach up to 75% previous pressure levels (18). Larger ureteral access sheath promotes lower intra-renal pressure. UAS (12/14Fr and 14/16Fr) reduced intra-renal pressure to safe levels while smaller access sheaths (9.5/11.5Fr) provided insufficient drainage due reduced diameter and therefore maintained elevated intra-renal pressures during ureteroscopy (15). Among medium size UAS (10/12Fr), data is not clear. Yoshida et al. tested four different brands of 10/12Fr UAS and only two of them were able to keep pressure under 40cmH2O (27).

In a retrospective case-control study to evaluated complications, patients who had underwent flexible ureteroscopy from 2008 to 2017 were compared in terms of use of UAS and postoperative infection. Fever rates were significantly lower in the 14/16Fr access sheath group when compared to those in 12/14Fr group (1.6% vs. 6.4%, p <0.001). Complications related to access sheath employment, such as ureteral wall lesion, were similar in both groups, probably due to preoperative stenting in this patient cohort (28). It seems conclusive that the use of a larger ureteral access sheath protects the renal colleting system from high pressures harms during ureteroscopy and consequently reduce infectious complications. However, a study developed by Traxer et al. described results from 359 procedures using a 12/14Fr UAS, showing an incidence of 13.3% of ureteral damage specially among men and elderly. Pre-stenting reduced this event by 7 times in this cohort (29). The American Urological Association (AUA) guidelines recommend the UAS use during flexible ureteroscopy for large stone treatment, to lower operative time and complications rates (30).

### Intra-renal Temperature

Holmium:yttrium-aluminum-garnet (Ho:YAG) laser is a worldwide spread technology for lithotripsy during flexible ureteroscopy. This is a highly efficient platform available to fragment all kidney and ureteral stone compositions (31, 32). Recently, there has been an interesting debate concerning ideal laser settings to apply for different scenarios during surgery and decision taking of whether to perform basketing or dusting techniques based on pulse width, frequency, and energy (33). Ho:YAG laser has a thermomechanical activity and the major concern about its use is about possible thermal effects on surrounding urothelium and renal tissue (34-36). Temperatures can reach 60°C after only 10 seconds of laser activation at 40W (36). Increments in intra-renal temperature can possibly lead to irreversible cellular damage by protein denaturation, in addition to cell’s genetic expression and composition damages, evolving to urothelial cellular death (37). Experimental study using a porcine model, using laser at 40W showed that short exposure of temperature above 40-60°C could promote tissue damage and cellular death (38). Studies showed temperatures above 43°C represent an increased risk for tissue damage and therefore should be avoided. Room temperature saline irrigation flow and suitable laser settings are paramount to maintain temperature in safe limit ranges (39). In an ex vivo experimental study, Molina et al. demonstrated the benefits of constant saline irrigation during ureteroscopy by reducing the temperature significantly during laser activation while using 10W total power (39). Wolin at al. developed another experimental model evaluating irrigation flow and laser settings. Without constant saline irrigation, all settings between 1.6 and 20W total power presented a temperature upper thermal damage threshold, and up to 100°C when a total power of 20W was applied. Safe temperature profile was observed when 100mL/min irrigation flow rate was applied. When reduced to 50mL/min, 40°C temperature measurement was detected only at 20W total power set (40). According to this study, we can conclude that constant irrigation flow is essential to thermal damage prevention during ureteroscopy. Aldoukhi et al. recently published data regarding irrigation flow, laser power setting and temperature. Authors concluded that higher irrigation flow rates are needed to use higher power settings to keep temperature in safety range (41). Different scenarios were created by Maxwell et al. to evaluate saline flow influence on temperature and once again results confirmed that, without irrigation, laser activation leads to potentially harmful temperature. When using an irrigation flow of 15mL/min and 40mL/min, temperatures remained at safe levels, except 15mL/min and 40W set, when a temperature of 60°C was observed. Temperature measurements at different locations in the collecting system yielded higher values at the ureter and renal pelvis. Caliceal temperature ranged from 36.5°C to 99°C when a constant saline irrigation flow of 5mL/min was applied (42). Butticè and cols. demonstrated that interrupted irrigation lead to rise temperatures up to thermal damage threshold regardless laser settings. Considering two distinct laser fiber diameters, no difference in results were observed. However, time needed to reach thermal damage temperature threshold was reduced in small diameters fibers and it was inversely proportional to energy pulse (35). As previously mentioned, instruments usage through working channel such as the laser fiber and baskets impair saline flow rate and can possibly contribute to a crucial reduction in irrigation rates increasing thermal cell injury. Therefore, close attention should be paid in high power settings, especially in impaired irrigation flow situation. Finally, Winship et al. studied pulse width effects of pulse and intra-renal temperatures in an in vitro model, utilizing flexible ureteroscope through a 11/13Fr ureteral access sheath with a 365-micron laser fiber. Long, short and modulated (Moses distance and contact) pulses were analyzed. Longer pulse promotes higher temperature, however within the safe range for cellular damage (43).

To date, there is no evidence that supports UAS use for reducing intra-renal temperature. However, it is an expert’s consensus that UAS use can improve saline inflow and outflow, preventing further intra-renal pressure elevation and thus keeping temperature at safety level.

An outbreaking technology for lithotripsy recently developed is promising to improve results compared to the Ho:YAG laser. Thulium fiber laser (TFL) presents unique characteristics, allowing urologists to use energy pulses as low as 0.025J, extremely high frequencies (up to 2000Hz), while using a thin silica fiber as small as 50μm (44). These properties enabled faster lithotripsy compared to the Ho:YAG systems with less stone retropulsion (45). Hardy et al. hypothesized a reduction in fiber diameter associated with better saline irrigation would contribute to reduce temperature patterns during laser activation. However, in an in vitro study, authors demonstrated a higher temperature increase with the TFL compared to the Ho:YAG platform and showed an even greater rise when using higher frequencies (45). In another in vitro study published by Peng et al., researchers demonstrated that total power and irrigation flow are the most important variables to affect temperature rise during TFL lithotripsy, establishing a safe threshold of 15mL/min and 20mL/min for 20w and 20-30W total power, respectively (46).

## CONCLUSION

Ureteroscopy is the most performed endourological procedure by urologists for ureteral and kidney stones treatment. Understand procedure details and particularities is cornerstone to obtain better results and decrease related complications. In this review we aimed to inform urologists about intra-renal pressure and temperature importance when performing flexible ureteroscopy based on the most up-to-dated available data.

Constant saline irrigation is critical to not exceed 40cmH2O threshold aiming to minimizing postoperative infections and perinephric fluid collections risks and other complications. Ureteral access sheath use seems to have a positive impact decreasing intra-renal pressure by promoting better kidney drainage.

Intraoperative temperatures can be increased by laser activation and irrigation plays an important role mitigating cellular thermal damage. Special attention should be paid using high power settings during lithotripsy once upon it can increase temperatures inside collecting system. Considering this review, the authors suggest some take home messages about this issue (Table-1).

**Table 1:** Take Home messages about intra-renal pressure and temperature for a safety ureterolithotripsy

**To keep intra-renal pressure in a safe threshold** - Saline irrigation during whole procedure - Saline bag elevated 60 cm above the surgical table - Maintain flow under gravity effect and use flushes carefully at specific times - Do not use pressurizer devices - Use ureteral access sheaths and observe output flow through it - Use urethral catheter during the procedure
**To keep intra-renal temperature in a safe threshold:** - Constant irrigation flow mainly during the laser activation - Increase the influx velocity when using high energy (above 20-40 W) - Maintain optimized flow mainly when performing lithotripsy inside the renal calix

In conclusion, saline irrigation is pivotal for visualization and reduction of intra-renal temperatures. It should be carefully used to avoid high intra-renal pressure related to increased postoperative complications risk.
